# Poor-Law Buildings and State Hospitals

**Published:** 1919-04-12

**Authors:** 


### The Hospital

The Workers' Newspaper of Administrative Medicine and Institutional Life, Administration, National Insurance and Health.

No. 1714, Vol. LXVI. SATURDAY, APRIL 12, 1919. PRICE TWOPENCE

### POOR-LAW BUILDINGS AND STATE HOSPITALS.

In our issue of February 15 we drew attention to the insufficiency of the accommodation in the London voluntary hospitals for the needs of the population. We pointed out that in the area of Greater London only 1.95 beds were available for every thousand of the population. In most of the provincial towns and country areas the proportion of hospital beds to population is much smaller. For years past the voluntary hospitals have been unable to cope with the numbers seeking admission to them. Many who should have been taken in have had to be treated as out-patients, or placed upon a waiting-list, or referred to poor-law infirmaries. It is notorious that the effect of the Insurance Act has been to increase the call for hospital beds. In the future it is certain that more and more hospital beds will be needed. There will be a larger demand for beds for the insured classes. The Ministry of Pensions will need for many years to come hospital accommodation for the treatment of its pensioners. The general public, which is realising more and more the difficulty, if not the impossibility, of adequately treating serious cases of illness, and more especially of cases needing surgical operation, in an ordinary house will insist upon its claims for fuller consideration. The extension of public-health work called for by the supporters of a Ministry of Health will lead to the discovery of much sickness at present insufficiently treated or not treated at all, sickness which in most cases it will be impossible to treat satisfactorily except in a hospital.

But it is not only a lack of hospital beds with which we are faced. Besides this, there is the fact that the voluntary hospitals have not kept in touch with the migrations of the population. In London most of our big hospitals are north of the Thames, and are collected within a comparatively small area. Some of them are placed in districts in which few people live. In the South and East of London there are large and densely populated areas without a single voluntary hospital within their bounds. This is true, too, of our large provincial towns. Speaking generally, it may be said that in the areas where the working classes live, where the need for hospital accommodation is greatest, there it is that the least provision has been made.

Here, then, is the problem the Ministry of Health must solve. Where is it to find the hospital beds needed now? Where is it to find the additional beds that will be needed very soon? How is it to find hospitals for areas in which no voluntary hospitals exist? How can it provide these hospital beds at the least possible expense? The answer to all these questions is, we believe, to be found in the poor-law infirmaries and workhouses. Practically in every parish throughout the length and breadth of Great Britain are institutions provided by Boards of Guardians for the sick and healthy under their care. Many of the Poor Law infirmaries are admirable examples of hospital construction. Most of them could easily and without great expense be made into good hospitals. Even the oldest and worst of them could be adapted so as to make admirable buildings for the treatment of chronic cases and of those needing the simpler forms of treatment. At present they are all insufficiently staffed with doctors and nurses, and inadequately equipped; but these are matters easy of rectification. The war has taught every one the fact, known long before to all those who studied our poor-law system, that the accommodation in these institutions was in excess of the requirements of the population served by them. But this is not all. Owing to the insufficiency of the medical and nursing staff, to the want of specialists, to the lack of equipment, many patients are inadequately treated and drift into a state of chronic invalidism. Even those patients who do receive adequate treatment are often kept an unnecessarily long time in infirmaries. There are no convalescent homes, there is no organised system of out-patient and home treatment to make early discharge possible.

The whole atmosphere of the infirmary differs from that of the voluntary hospital. In the latter the pressure upon the beds leads to the discharge of the patient so soon as he no longer needs treatment in bed and constant skilled supervision. In the former there is no such stimulus, and patients are kept long after this stage is reached. For this the absence of an out-patient department and the fact that the district medical officers have no direct connection with the infirmary are largely responsible. But there are other reasons. Owing to the smallness of the staff many patients are kept to help in the domestic work in the wards, and the feeling that an infirmary should combine the functions of a home of rest with those of a hospital has the result that in many infirmaries patients are seldom sent out until they ask for their discharge. If the infirmaries were properly staffed and equipped, if they were made centres for out-patient and home treatment, and trained nurses were provided for nursing patients in their own homes, if all the infirmaries in large areas were linked up together and specialised, if full use were made of every bed, and if convalescent homes were provided, the infirmaries could easily treat at least three times the number of patients that pass through them under present conditions.

But before the infirmaries can be used to meet the increasing demands of the community for hospitals they must be dissociated from the Poor Law. The entry to the poor-law infirmary is restricted by a procedure the self-respecting poor detest. The name of poor-law infirmary is associated with a stigma that keeps many sorely in need of its help from its gates. Nothing but a radical change in its constitution will overcome this antagonism. The London Labour Party supports this view strongly. In a manifesto to the people of London on the recent election of Guardians, it says that Labour takes the view that the whole Poor-Law system is bad and must be abolished.

The poor-law infirmary must be taken from the Guardians and become a State hospital linked up directly to the Ministry of Health or indirectly to it through the County Councils. Nothing short of this will make it acceptable to the general public and lead them to avail themselves of its services.

